# Mesenchymal stem cell exosome-derived miR-223 alleviates acute graft-versus-host disease via reducing the migration of donor T cells

**DOI:** 10.1186/s13287-021-02159-2

**Published:** 2021-02-26

**Authors:** Weijiang Liu, Na Zhou, Yuanlin Liu, Wei Zhang, Xue Li, Yang Wang, Rongxiu Zheng, Yi Zhang

**Affiliations:** 1 Department of Experimental Hematology and Biochemistry, Beijing Institute of Radiation Medicine, 27 Taiping Road, Beijing, 100085 People’s Republic of China; 2 Department of Pediatrics, Tianjin Medical University General Hospital, 154 Anshan Road, Tianjin, 300052 People’s Republic of China; 3 Department of Medical Administration, The Sixth Medical Center of PLA General Hospital, Beijing, 100048 China

**Keywords:** Mesenchymal stem cells, miR-223, ICAM-1, Acute graft-versus-host disease

## Abstract

**Background:**

Mesenchymal stem cells (MSCs) have been utilized in treating acute graft-versus-host disease (aGvHD) as they show strong immunosuppressive capacity through the release of various mediators, including immunosuppressive molecules, growth factors, chemokines, and exosomes. MicroRNAs (miRNAs) derived from MSC exosomes (MSCs-Exo) play a critical role in the regulation of immune responses. However, the function of miRNAs in treating aGvHD remains unknown. Here, we performed expression profiling of exosome-miRNAs from human umbilical cord MSCs (huc-MSCs) and murine compact bone MSCs (mb-MSCs) to investigate their immunoregulation effects in aGvHD.

**Methods:**

Huc-MSCs-Exo and mb-MSCs-Exo were isolated and constructed MSCs-Exo-derived miRNA expression profiling using high-throughput sequencing. High expression of miR-223 was identified in both kinds of MSCs-Exo by bioinformatics analysis and quantitative real-time PCR (qPCR). In vitro cell crawling assay, transmigration assay and adhesion assay were subsequently applied to investigate the regulation of miR-223 on T cells. MiR-223 target gene was analyzed by western blot, luciferase analysis, and qPCR. Moreover, murine aGvHD model was established by infusing splenocytes and bone marrow nuclear cells from C57BL/6j mice (H-2Kb) into BALB/c recipient mice (H-2Kd). For therapeutic effect, MSCs or miR-223 Agomir were injected via tail vein. The general conditions of the mice in each group were monitored. Hematoxylin-eosin (H&E) staining was used to detect pathological changes of mice spleen, liver, and intestine. Mechanistically, immunofluorescence and flow cytometry were used to evaluate donor T cell migration, and enzyme-linked immunosorbent assay (ELISA) was used to detect the expression of serum inflammatory cytokines IFN-γ, TNF-α, and IL-17.

**Results:**

High-throughput sequencing revealed high expression of miR-223 in huc-MSCs-Exo and mb-MSCs-Exo. MiR-223 could restrain adhesion and migration of T cells by inhibiting ICAM-1 expression in mouse lymphatic endothelial cells. MiR-223Agomir infusion attenuated aGvHD clinical symptoms, reduced the donor T cell infiltration into the spleen, liver, and intestine, and decreased inflammatory cytokines IFN-γ, TNF-α, and IL-17.

**Conclusion:**

MSCs-Exo-derived miR-223 could attenuate aGvHD in mice through decreasing donor T cell migration. Our results unveil a new role of MSCs-Exo containing miR-223 in the treatment of aGvHD.

**Supplementary Information:**

The online version contains supplementary material available at 10.1186/s13287-021-02159-2.

## Background

Acute graft-versus-host disease (aGvHD) is a severe autoimmune condition caused by immune responses from allogeneic T cells during hematopoietic stem cell transplantation [1–3]. To date, several studies indicated that mesenchymal stem cells (MSCs) attenuated aGvHD through suppressing the proliferation and activity of T cells, inducing the regulatory CD4^+^CD25^+^ T cells (Treg cells) and inhibiting the proliferation and activation of antigen-presenting cells (APC) such as dendritic cells [4–6]. Nevertheless, the mechanisms remain unclear about the regulation of MSCs on these cells.

Mesenchymal stem cells (MSCs), acting as a pleiotropic immune regulator, are reported to suppress immune processes, in which nearly all immune cells including T cells, natural killer (NK) cells, B cells, and dendritic cells (DCs) are affected [4, 5, 7–9]. In the recent years, more attention has been paid to potential efficiency of MSCs in aGvHD as they show low toxicity and high expansion in vitro [6, 10]. The immunosuppressive capacities of MSCs are not continuous but were licensed by inflammatory cytokines within the microenvironment [11–13]. In fact, different states of inflammation can result in significantly different responses to MSCs treatment, which indicates the plasticity of immunomodulation by MSCs. Thus, tissue location and inflammatory status are critical determinants of the immunoregulatory properties of MSCs and might affect the therapeutic potential of these cells in inflammatory diseases. However, the plasticity of MSCs mediated immunoregulation molecular mechanisms are still unclear, which is a limit of MSCs in treating inflammatory diseases.

The immunoregulatory mechanism of MSCs involves the autocrine or paracrine secretion of factors, chemokines, and exosomes [5, 14]. At present, several studies have shown that exosomes can simulate almost all the biological functions of MSCs [15–18]. Intramembrane DNA, RNA, lipids, proteins, and non-coding RNA of the exosomes participate in the regulation of inflammatory responses [19, 20]. MicroRNAs (miRNAs) play a critical role in the regulation of immune responses. Previous studies had demonstrated that several miRNAs (e.g., miR-181c, let-7b, and miR-146b) in the MSCs exosomes (MSCs-Exo) could suppress the inflammatory responses caused by innate and adaptive immunity [21–23]. Meanwhile, these exosomes can be absorbed by neighboring or distant cells to modulate the function of recipient cells [17, 19]. These led to a hypothesis that MSCs may secrete exosomal miRNAs, which served as extracellular molecules involving in regulating MSC immunological functions and potential therapeutic agents for aGvHD. Furthermore, unlike MSCs, MSCs-Exo containing miRNA-modulated immunosuppression are independent on inflammatory cytokines, to reduce inflammation in treating inflammatory diseases. These anti-inflammatory miRNAs could be applied to replace the MSCs injection as a “free cell therapy,” which is easier to observe and control the therapeutic effect.

To explore the therapeutic effects of MSCs-Exo-derived miRNA on aGvHD, we constructed a miRNA landscape of human umbilical cord and murine compact bone-derived MSCs-Exo by high-throughput sequencing, which showed high miR-223 expression. Previous studies demonstrated that miR-223 showed effects on macrophage differentiation, hematopoietic differentiation, and viral infection, and serve as a negative feedback mechanism controlling excessive innate immune responses in the maintenance of myeloid cell homeostasis [24–26]. However, little is known about the immunosuppressive capacities of MSCs-Exo-derived miR-223 exert in aGvHD environment. In this study, we showed miR-223Agomir could attenuate the aGvHD progression by reducing donor T cell migration and inflammatory response. These results revealed a novel role of miR-223 in regulating the immunomodulatory properties of MSCs in aGvHD.

## Materials and methods

### Mice

Male BALB/c mice served as the recipient mice [8-week-old, specific pathogen free (SPF)]. The donor mice were 6-week-old SPF-grade female C57BL/6j mice (H2kb). Mice were purchased from Beijing Vital River Laboratory Animal Technology [license SCXK (Beijing) 2016-0001]. All the mice were reared in SPF animal rooms in the Experimental Animal Center at the Beijing Academy of Military Medical Sciences. The recipient mice were fed with sterilized food and water containing gentamicin (320 mg/L) and erythromycin (250 mg/L). All experiments in this study were performed in accordance with the Beijing Academy of Military Medical Sciences Guide for Laboratory Animals.

### Cell preparation

Human umbilical cord MSCs (huc-MSCs) were isolated and cultured as follows: human umbilical cords (huc) were collected from normal full-term pregnancies according to the regulations of the Research Ethics Committee of Jishuitan Hospital (Beijing, China). After washing with phosphate buffered saline (PBS) to remove residual blood under sterile conditions, whole UCs were shredded into small pieces of 2 mm^3^. The tissues were digested in 0.1% type II collagenase at 37 °C for 40 min and transferred the digested pieces into alpha-MEM containing 10% fetal bovine serum (FBS), 100 U/ml penicillin, and 100 mg/ml streptomycin. Medium was changed every 2 to 3 days. When the adherent cells reached a confluence of approximately 80%, cells were collected by using 0.25% trypsin for culture expansion and identification.

Murine compact bone MSCs (mb-MSCs) were isolated and cultured as follows: Femur and tibia from SPF-grade female C57BL/6j mice (1 week) were collected and shredded into small pieces. The small bone fragments were digested using type II collagenase at 37 °C for 45 min and transferred the digested bone fragments into alpha-MEM containing 10% fetal bovine serum (FBS), 100 U/ml penicillin, and 100 mg/ml streptomycin. Medium were changed every 2 to 3 days. Upon a confluence of 80–90%, cells were subcultured at a ratio of 1:3. Cells at passages 3 were used to further identification.

### Differentiation assay of MSCs

To identify the adipogenic differentiation capacity, cells (8 × 10^4^) were cultured with alpha-MEM containing 10% FBS with 10^− 3^ mM dexamethasone, 0.5 mM isobutyl methylxanthine, 0.2 mM indomethacin, and 10 μg/ml insulin (Sigma, Germany) for 2 weeks. To demonstrate the presence of adipocytes, cytoplasmic inclusions of neutral lipids were stained with Oil Red O (Sigma, Germany).

Osteogenic differentiation capacity was assessed by incubating the cells (7 × 10^3^) with alpha-MEM containing 10% FBS with 10^− 7^ mM dexamethasone, 0.5 mM ascorbic acid, and 10 mM β-glycerol phosphate (Sigma, Germany) for 2 weeks. Osteoblasts were identified by immunocytochemical stain with alkaline phosphatase (ALP). These detail methods were according to the previous description [27, 28].

Bone marrow cells and splenocytes were isolated from 6-week-old C57BL/6j mice. Mice were euthanized using carbon dioxide, followed by obtaining the spleen and bone marrow tissues. Erythrocytes were lysed by using erythrocyte lysis buffer. Fresh splenocytes and bone marrow were obtained and washed twice with PBS. These cells were used for transplantation into the irradiated recipient mice.

For the splenocyte staining, the splenocytes for transplantation were labeled with CellTracker™ CM-DiI dye (C7000, Invitrogen, Waltham, MA, USA). This was achieved by adding CM-Dil (2 μl) to a splenocyte suspension and incubating at 37 °C in the dark for 5 min, followed by incubating at 4 °C in dark for 15 min. Then, splenocyte suspension was resuspended in PBS and centrifuged (250*g*, 5 min) to remove the unbound dye.

### Flow cytometry analysis

Human umbilical cord-derived adherent cells were phenotypically characterized by flow cytometry. Phycoerythrin (PE)-conjugated monoclonal antibodies against mouse CD73 (12-0739-42), CD105 (12-1051-82), and CD90 (12-0909-42) and fluorescein isothiocyanate (FITC)-conjugated antibodies against mouse CD34 (11-0341-82), CD45 (11-0451-82), and CD14 (11-0149-42, eBioscience, Waltham, MA, USA) were used.

For intercellular cytokine staining, splenic lymphocytes were collected and stained with anti-mouse PE-CD4 (12-0041-82), anti-mouse PE-CD8 (12-0081-82), and allophycocyanin-conjugated antibodies against mouse (APC) H2kb (17-5958-82) for 30 min at 4 °C. Th1 cells were stained with anti-mouse FITC-CD3 (11-0032-80) and anti-mouse PE-CD4 (MCD0404) for 30 min at 4 °C. Cells were then fixed, permeabilized, and stained for transcription factors using the Fixation/Permeabilization Diluent (00-5223-56) according to the manufacturer’s instructions. For cytokine staining, the cells were stained with anti-mouse APC-IFN-γ (47-7311-80) and APC-IL-4 (17-7041-82) (eBioscience, Waltham, MA, USA) for 30 min at 4 °C. Signals were recorded by flow cytometry with a FACScalibur system (Becton Dickinson), and data were analyzed with the FlowJo software.

### Exosome purification and characterization

Huc-MSCs and mb-MSCs at passage 3 were cultured in serum-free culture medium. In the presence of a cellular density of 80%, the supernatant was collected and centrifuged at 700*g* for 10 min to remove cell debris. Centrifugation was then applied to the medium at 9000*g* at 4 °C for 30 min, and supernatant was collected again. Exosomes were isolated by ExoEasy Maxi kit (76064, Qiagen, Dusseldorf, Germany) and resuspended in PBS. The characterization of exosomes was confirmed by measuring expression of exosome-specific markers TSG101 and CD63 by Western blot analysis and particle size by NanoSight analysis (RiboBio, China). The concentration of exosomes was determined by analyzing protein concentration using the Bio-Rad protein quantitation assay kit (5000001, Bio-Rad, Hercules, USA) with BSA as a standard.

### Electron microscopy

For electron microscopy, exosomes were fixed with 2% paraformaldehyde and loaded on 200 mesh formvar and carbon-coated copper grids which had been glow discharged for 15 s. Samples were incubated on grids for 30 s and subsequently stained with a 2% uranyl acetate solution. Grids were viewed using a JEOL 1200EX II (JEOL) transmission electron microscope and photographed using a Gatan digital camera (Gatan).

### MSCs-exosomes contain functional miR-223

MSCs (1 × 10^6^) were injected into C57BL/6j mice (*n* = 5) via the tail vein. The mice in the control group were injected with the same volume of PBS (*n* = 5). After 48 h treatment, the peripheral blood in recipient mice was collected and centrifuged at 3000*g* for 15 min. The serum was harvested, and serum exosomes were isolated according to the manufacturer’s instructions (76064, Qiagen, Dusseldorf, Germany).

### Transient transfection experiment

Human umbilical vein endothelial cells (HUVECs) or mouse primary lymphatic endothelial cells (mLECs; C57-6092, Cell Biologics, Chicago, USA) were seeded into 24-well plates (2 × 10^5^/well) and cultured using complete RPMI 1640 medium containing 10% FBS. Upon a cell density of 50–70%, miR-223 mimic (100 nM) and negative control was separately transfected with jetPrime transfection reagent (114–15, Polyplus, France). Cells were collected after 48 h. Then qPCR and Western blot were used to measure expression of miR-223 and target gene *ICAM-1*.

### Exosomes transport miR-223 into the HUVECs

HUVECs (2 × 10^5^) were seeded into each well in 6-well plates. The exosomes (2 μg/well) were added to the experimental group, and an equal volume of PBS buffer was added to the control group as previously described [29]. The cells were cultured in RPMI 1640 medium containing 10% FBS for 24 h. The supernatant was discarded, and the cells were washed twice with PBS to remove residual exosomes. Finally, 0.25% trypsin was used for digestion, followed by cell collection. Afterwards, qPCR and Western blot were used to measure the expression of miR-223 and ICAM-1.

### Luciferase analysis

The 293T cells were transfected using jetprime with pGL3-ICAM-1-3′UTR plasmid, Co-reporter vector pRL-TK (containing renilla), and miR-223 mimic (100 nM). After 48 h, the supernatant of the culture system was discarded, and cells were washed with PBS. The cells were lysed using the passive lysis buffer from the Dual-Luciferase® Reporter Assay System (E1910, Promega, Wisconsin, USA), and luciferase activity was measured according to the manufacturer’s instructions.

### Th1 cell differentiation in vitro

CD4^+^ Th1 cell differentiation was induced according to the previous study [30]. Briefly, mice spleen was homogenized and filtered through a cell strainer (40 μm). Erythrocytes were lysed using erythrocyte lysis buffer. CD4 microbeads (Miltenyi Biotech, L3T4, Germany) were used to isolate CD4^+^ T cells, which were cultured in a 24-well plate coated with anti-CD3ε (3 μg/ml, 145-2C11, Biolegend, CA, USA) and anti-CD28 (5 μg/ml, 102115, Biolegend, CA, USA). The culture medium contained IFN-γ (20 ng/ml, 505701, Peprotech, NJ, USA), IL-12 (5 ng/ml, Peprotech, 505202), and anti-IL4 (5 μg/ml, Biolegend, 504101, CA, USA). Upon cell culture for 3–5 days, flow cytometry was used to measure the expression of IFN-γ and IL-4.

### Th1 cell staining

The cells were suspended gently in CellTracker™ (1:1000, C7025, Invitrogen, Waltham, MA, USA) staining solution. The mixture was incubated at 37 °C for 30 min and centrifugated (250*g*, 5 min) to remove the CellTracker™ Working Solution. The stained cells were resuspended with culture medium and used for the following assays.

### Cell crawling assay

C57BL/6j mLECs were seeded into coated channeled chamber slides (μ-Slide VI^0.4^,1709291, IBIDI, Germany). Monolayers were transfected with miR-223 mimic and normal control until 24 h, and then stained Th1 cells (3 × 10^4^) were added. About 20 min later, chambers were rinsed twice to remove non-adherent Th1 cells. After a further 10 min equilibration at 37 °C, time-lapse imaging was performed on Operetta CLS™ (PerkinElmer Operetta CLS). The images were analyzed using Operetta primarily software.

### Transmigration assay in vitro

The mLECs were transfected with miR-223 mimic or negative control for 24 h and seeded into the upper side of transwell chamber (#PIMP12R48, Millipore, Massachusetts, USA), cultured until confluence. Then, stained Th1 cells (5 × 10^4^) were added in the upper transwell chamber. After 4 h, medium in the bottom well was collected and the number of stained Th1 cells was quantified by cell counter (Luna, UK).

### Adhesion assay in vitro

The miR-223 mimic or normal control were transfected into mLECs for 24 h and seeded into a coated 96-well clear bottom (Corning Costar®). Then Th1 cells (1 × 10^4^) were added. Non-adherent cells were collected and the number of Th1 cells was measured by cell counter 45 min later.

### Construction of mouse aGvHD model

BALB/C mice (8-week-old) were irradiated with a single dose of 800 cGy total body irradiation (TBI, Co60γ source). The aGvHD mice were infused with nucleated bone marrow cells (1 × 10^7^) and splenocytes (1 × 10^7^) isolated from C57BL/6j mice (6-week-old) through tail vein injection. After 24 h of irradiation, MSCs (5 × 10^5^/mouse, *n* = 15), chemically synthesized miR-223Agomir and micrON™ Ago NControl#22 (RiboBio, China) (10 nmol/mouse, n = 15) were used to treat aGvHD mice.

The miR-223Agomir or micrON™ Ago NControl#22, serving as the negative control, were centrifuged transiently to achieve miRNA powder aggregation at the bottom of the centrifugation tube. Subsequently, PBS buffer (1 ml) was added to dissolve the powder. The solution was injected into recipient mice via tail vein 1 day before irradiation, and 1, 4, and 7 days after irradiation.

### Western blot

Cells were lysed with lysis buffer (87787, Thermo Scientific™, USA) to isolate proteins. The protein samples (20 μg) were subsequently separated via 12% SDS-PAGE, and then were transferred onto 0.45 μm PVDF membranes. PVDF membrane blots were blocked in 5% milk in Tris-buffered saline with Tween 20 (TBS-T) for 1 h at room temperature and incubated overnight at 4 °C with Rabbit Anti-TSG101 (ab125011, Abcam, Massachusetts, UK), Rabbit Anti-CD63 (Ab217345, Abcam, Massachusetts, UK), and Mouse anti-ICAM-1 (sc-8439, Santa Cruz Biotechnology, Delaware Ave, USA). Afterwards, the mixture was incubated with HRP-conjugated secondary antibodies in blocking solution for 1 h at room temperature. Finally, the protein bands on blots were detected with the SuperSignal™ West Femto Maximum Sensitivity Substrate (34095, Thermo Fisher, Waltham, MA, USA), which contains Working Solution mixed with equal parts of the Stable Peroxide Solution and the Luminol/Enhancer Solution. Protein bands were analyzed using Image Lab software (BioRad, Hercules, USA).

### ELISA

Serum IFN-γ, TNF-α, and IL-17 were measured using IFN-γ Mouse enzyme-linked immunosorbent assay (ELISA) Kit (BMS6027TEN, Invitrogen, Waltham, MA, USA), TNF-α Mouse ELISA Kit (BMS607HS, Invitrogen, Waltham, MA, USA), IL-17 Mouse ELISA Kit (BMS6001, Invitrogen, Waltham, MA, USA)), respectively. All protocols were conducted according to the manufacturer’s instructions.

### Quantitative real-time PCR

Total RNA was extracted from exosomes and cells using Trizol reagent (15596018, Invitrogen, Waltham, MA, USA)). The cDNA synthesis was conducted using the commercial reverse transcription kit (CW2020, CWBIO, Beijing, China). The stem-loop method was used for reverse transcription of miRNA. Then qPCR was carried out using the UltraSYBR One-Step Kit (CW2624, CWBIO, Beijing, China) according to the manufacturer’s instructions. PCR amplification was conducted on the 7500 Real-Time system with GAPDH serving as a reference gene. The primer sequences used for qPCR are listed in Table 1.

**Table 1:** Primer sequence

Gene	Primer sequence (5′–3′)
miR-223	ACACTCCAGCTGGGTGTCAGTTTGTCAA
Forward	
miR-223	CTCAACTGGTGTCGTGGAGTCGGCAATTCAGTTGAGTGGGGTAT
Reverse	
snoRNA202	ACACTCCAGCTGGGGCTGTACTGACTTGATG
Forward	
snoRNA202	CTCAACTGGTGTCGTGGAGTCGGCAATTCAGTTGAGCATCAGAT
Reverse	
URP	TGGTGTCGTGGAGTCG
Human ICAM Forward	ACCATCTACAGCTTTCCGG
Human ICAM Reverse	ACACTTCACTGTCACCTCG
Mouse GAPDH Forward	ACTCTTCCACCTTCGATGC
Mouse GAPDH reverse	CCGTATTCATTGTCATACCAGG
Human GAPDH Forward	TCAAGATCATCAGCAATGCC
Human GAPDH Reverse	CGATACCAAAGTTGTCATGGA

### Statistical analysis

Data were presented as mean ± standard error of mean. When comparing two groups with a sample size of ≥ 3, an unpaired two-tailed Student’s *t* test was used. Mann–Whitney nonparametric tests were used to compare two independent groups with a sample size of ≥ 3. When data were paired and sample size was ≥ 3, Wilcoxon matched-pairs tests were used. One-way ANOVA were used to compensate for multiple testing procedures. Data analysis was performed using GraphPad Prism Version 6.0 software. A value of *P* < 0.05 was considered to be statistically significant.

## Results

### Isolation and definition of exosomes from human and murine MSCs

**Fig. 1:**
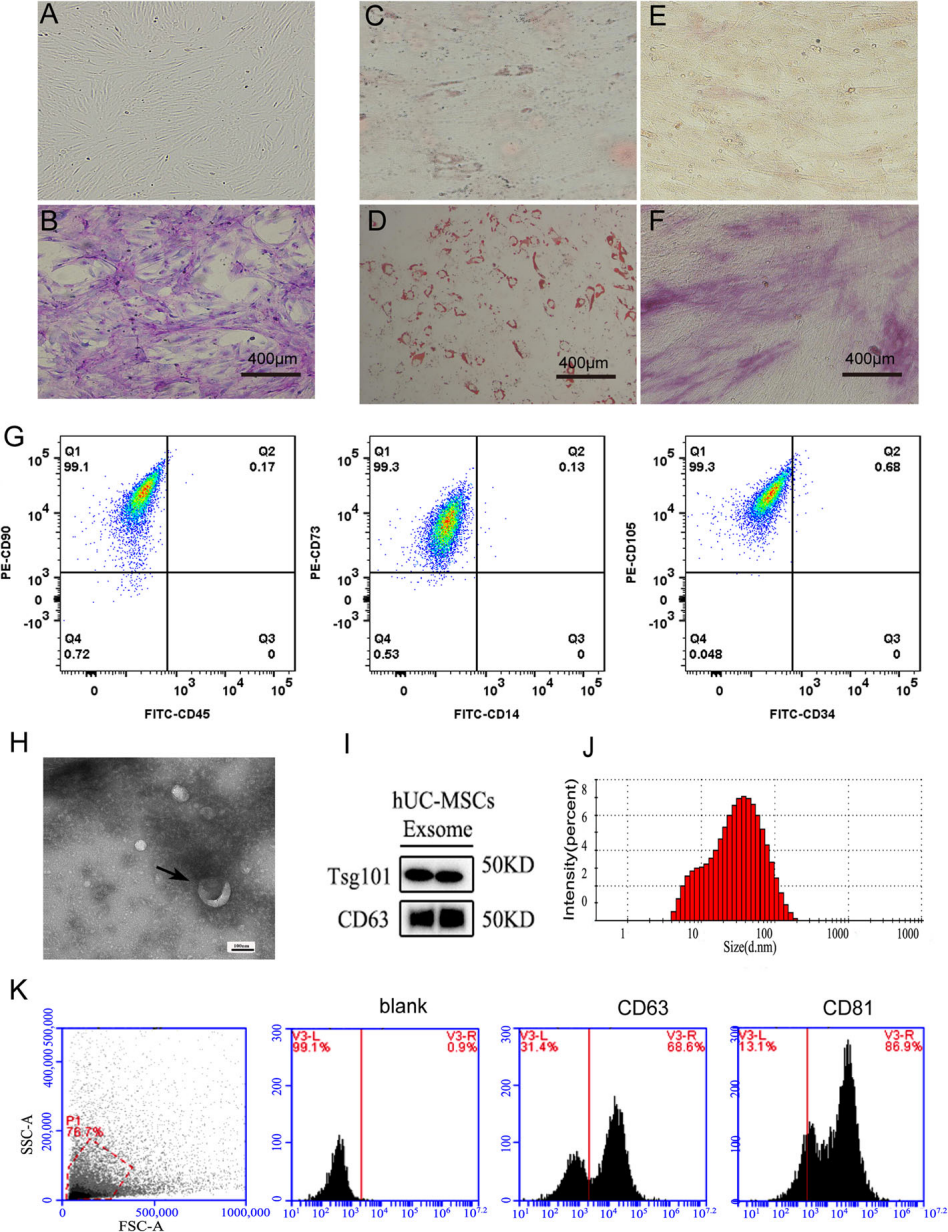
Identification of human umbilical cord mesenchymal stem cell (huc-MSCs)-derived exosomes. Nucleated cells isolated from umbilical cord displayed a fibroblast-like morphology (**a**). The adherent cells (P3) were stained with Wright-Giemsa (**b**). Adipogenesis differentiation was indicated by the presence of lipid drops that stained with Oil Red O (**c** and **d**; **c** control; **d** experimental group). Osteogenic differentiation was shown by intracytoplasmic accumulation of alkaline phosphatase (**e** and **f**, **e** control; **f** experimental group). The phenotype of huc-MSCs was detected by flow cytometry. CD45, CD34, and CD14 were negative while CD73, CD105, and CD90 were positive (**g**). huc-MSCs-Exo (marked by the black arrows) were observed under electron microscopy (**h**). Exosome-specific markers (e.g., TSG101 and CD63) were positive by Western blot analysis (**i**). NanoSight analysis indicated that particle size of MSCs-Exo was 30–100 nm (**j**). Analysis of exosome-specific markers CD81 and CD63 was positive by flow cytometry (**k**)

We first isolated and identified MSCs from human umbilical cord as conventionally described. Human umbilical cord nucleated cells separated by density gradient displayed fibroblast-like morphology (Fig. 1a, b). As shown in Fig. 1c–f, adipogenic differentiation of adherent cells was indicated by accumulation of Oil Red O staining lipid-rich vesicles. In the osteogenic culture system, the adherent cells displayed strong alkaline phosphatase activity. These results confirmed the multipotency of MSCs. The immunophenotype of the human umbilical cord adherent cells was determined by flow cytometry. The culture-expanded adherent cells were positive for CD73, CD90, and CD105 and were negative for CD14, CD34, and CD45 (Fig. 1g). All results presented herein indicated that the adherent cells isolated from human umbilical cord were MSCs. The characteristics of mb-MSCs were similar to that of huc-MSCs (Fig. S1A-S1G).

Sequentially, we identified huc-MSCs-derived exosomes. Electron microscopy (Fig. 1h) and NanoSight analysis (Fig. 1j) revealed that particles isolated by centrifugation contain abundant huc-MSCs-Exo with a diameter of 30–100 nm. Expression of exosome-specific markers (i.e., TSG101 and CD63) was confirmed by Western blot (Fig. 1i). In addition, flow cytometry results showed that expression of other exosome-associated protein markers (i.e., CD81 and CD63) was positive (Fig. 1k). The characteristics of mb-MSCs-Exo were similar with huc-MSCs-Exo (Fig. S1H-S1J).

### MiR-223 highly expressed in both huc-MSCs-Exo and mb-MSCs-Exo

**Fig. 2:**
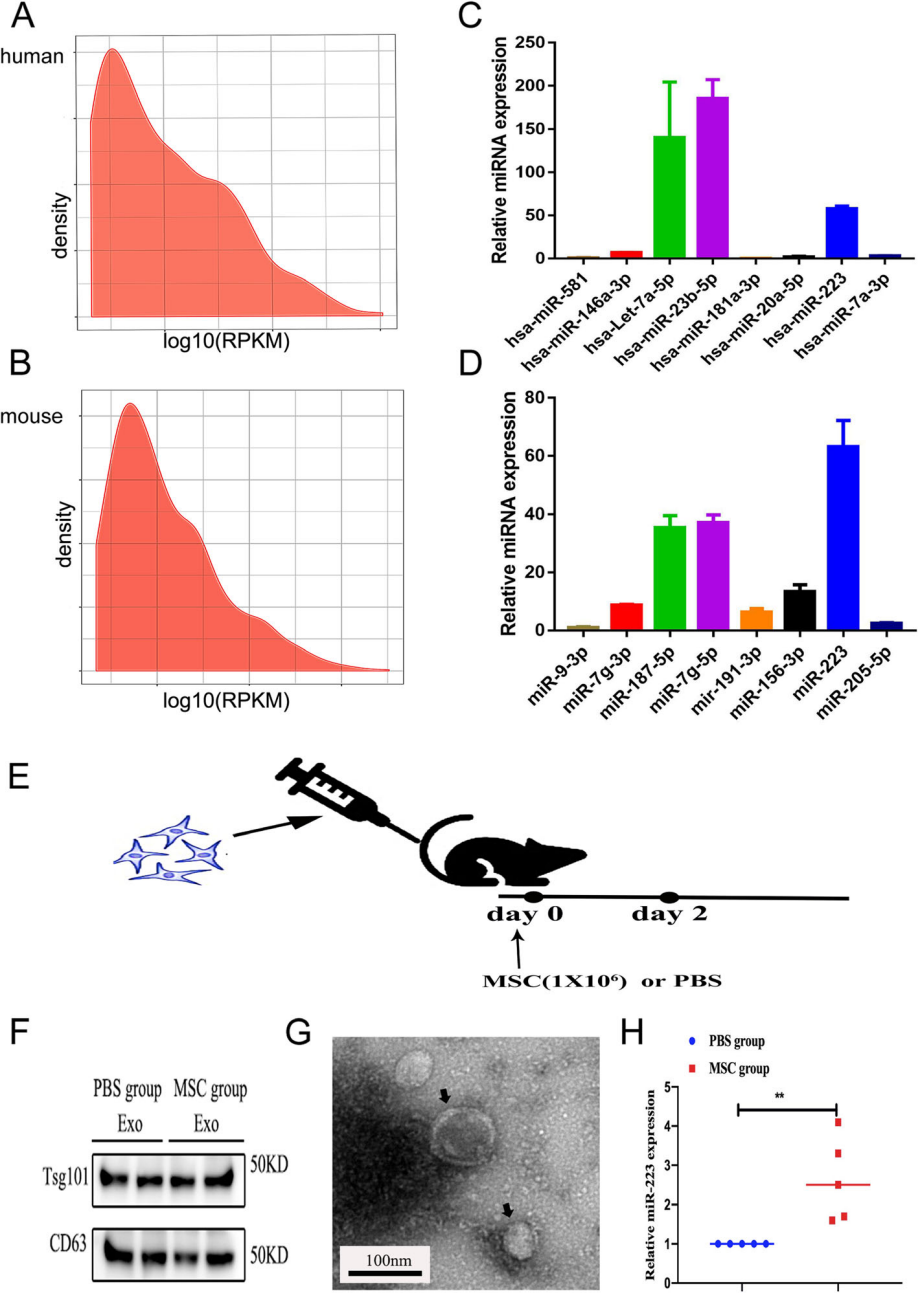
MSCs-Exo containing miR-223. MiRNA expression spectrum in huc-MSCs-Exo (**a**) and mb-MSCs-Exo (**b**) were analyzed by high-throughput sequencing. High expression of miR-223 in huc-MSCs-Exo (**c**) and in mb-MSCs-Exo (**d**) was measured by qPCR. MSCs (1 × 10^6^/dose) were injected into C57BL/6j mice. About 48 h after injection, the serum-derived exosomes were isolated, and the expression of miR-223 was tested by qPCR (**e**). Western blot analysis of serum exosome-specific markers TSG101 and CD63 was positive (**f**). Exosomes secreted by mb-MSCs (marked by the black arrows) were observed under electron microscopy (**g**). The qPCR indicated the high expression of miR-223 about 48 h after injection (**h**). Data were presented as mean ± SEM. Measured in three independent experiments, *n* = 5 per group. **P < 0.01

To explore the miRNA expression spectrum of huc-MSCs-Exo and mb-MSCs-Exo, high-throughput sequencing was used to identify miRNAs of huc-MSCs-Exo and mb-MSCs-Exo. The result showed that miR-223 was highly expressed in both huc-MSCs-Exo and mb-MSCs-Exo (Fig. 2a, b). The qPCR supported the sequencing data (Fig. 2c, d). Furthermore, to determine whether MSCs-Exo contained functional miR-223 in vivo, mb-MSCs (1 × 10^6^/dose) were injected into C57BL/6j mice (Fig. 2e). After 48 h, the serum-derived exosomes were isolated and observed by electron microscopy (Fig. 2g). Western blot analysis confirmed the expression of serum exosome-specific markers (i.e., TSG101 and CD63) (Fig. 2f). Moreover, qPCR was used to measure the expression of miR-223 in the mb-MSCs-Exo. The result showed high expression of miR-223 (Fig. 2h). Taken together, these data confirmed the high expression of miR-223 in MSCs-Exo and functional in vivo.

### MSCs-Exo-derived miR-223 inhibited the expression of the target gene ICAM-1

**Fig. 3:**
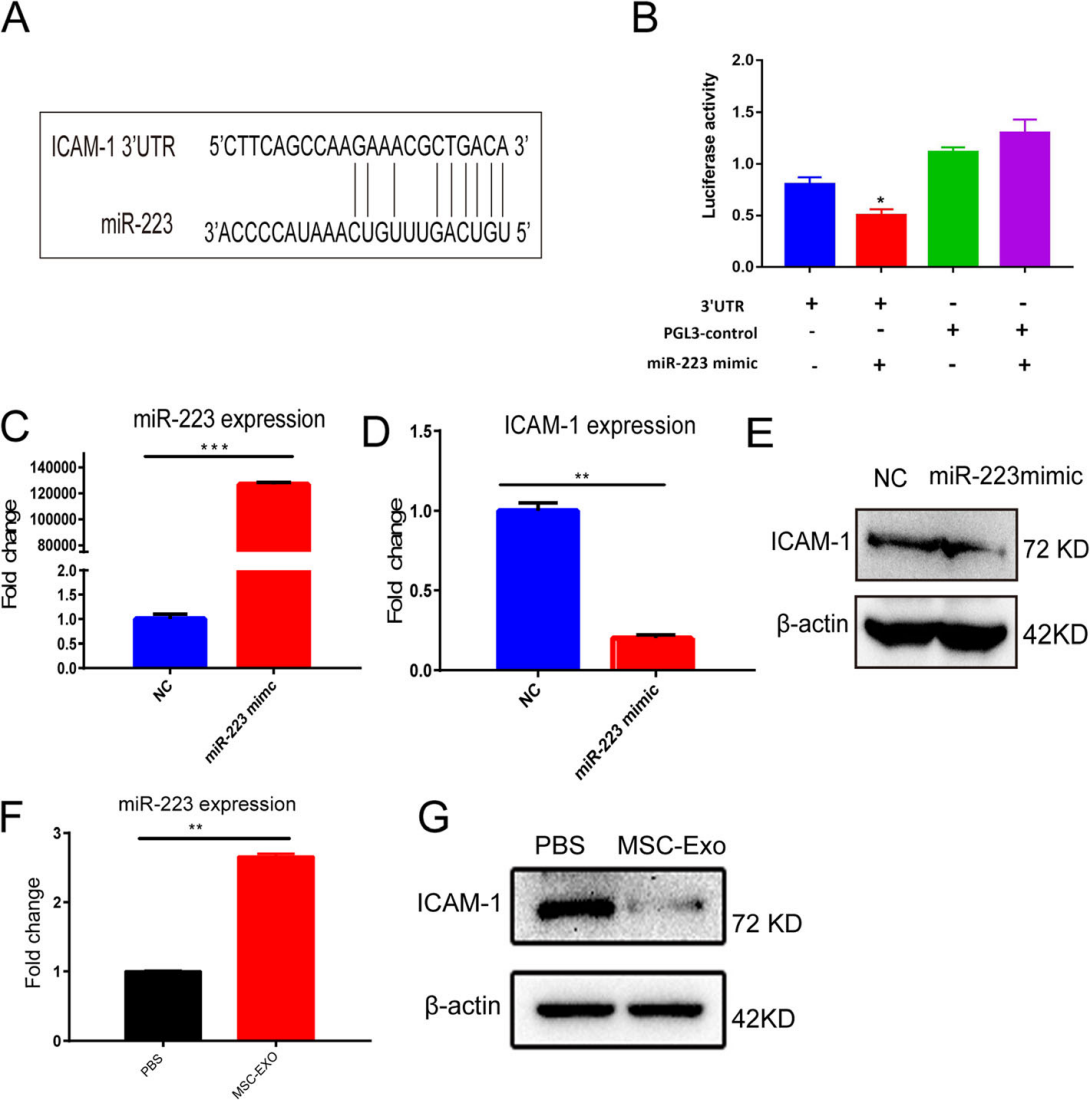
MSCs-Exo-derived miR-223 inhibited ICAM-1 expression. Bioinformatic analysis indicated that miR-223 regulated the expression 3′-UTR of ICAM-1 (**a**). Transfection of miR-223 mimic induced significant downregulation of ICAM-1 (**b**). High expression of miR-223 by qPCR after transfection of 100 nM miR-223 mimics (**c**). ICAM-1 mRNA and protein expression showed decrease as revealed by qPCR and Western blot (**d**, **e**). After 24 h co-culturing of MSCs-Exo (2 μg) with HUVECs, the expression of ICAM-1 in HUVECs showed decrease as revealed by qPCR (**f**) and Western blot (**g**). Data were presented as mean ± SEM. Measured in three independent experiments. *P < 0.05, ***P < 0.001

We performed bioinformatics assays (miRase, NCBI, and TargetScan) to predict the potential targets of miR-223. Among the candidates, we found IACM-1 was worthy for further study since it was associated with T cell migration (Fig. 3a). To investigate whether ICAM-1 was the target gene of miR-223, we simultaneously transfected ICAM-1-3′UTR luciferase reporters and miR-223 mimic into HEK 293 cells. The results showed that miR-223 mimic transfection could significantly reduce luciferase activity (Firefly/Renilla) in HEK293T cells (Fig. 3b, *P* < 0.05).

To further determine the regulatory effects of miR-223 on ICAM-1, we transfected miR-223 mimic into HUVECs. Both qPCR and Western blot results indicated that there was a significant downregulation of ICAM-1 in mRNA and protein levels (Fig. 3c–e). Moreover, MSCs-Exo (2 μg) was co-cultured with HUVECs for 24 h to verify whether exosomes could transport miR-223 into the target cells to inhibit target gene expression. The result showed that miR-223 expression was upregulated in HUVECs, and ICAM-1 protein expression was downregulated (Fig. 3f, g). These data suggested that miR-223 derived from MSCs-Exo inhibited the expression of the target gene *ICAM-1*.

### MiR-223 restrained T cell adhesion and migration

**Fig. 4:**
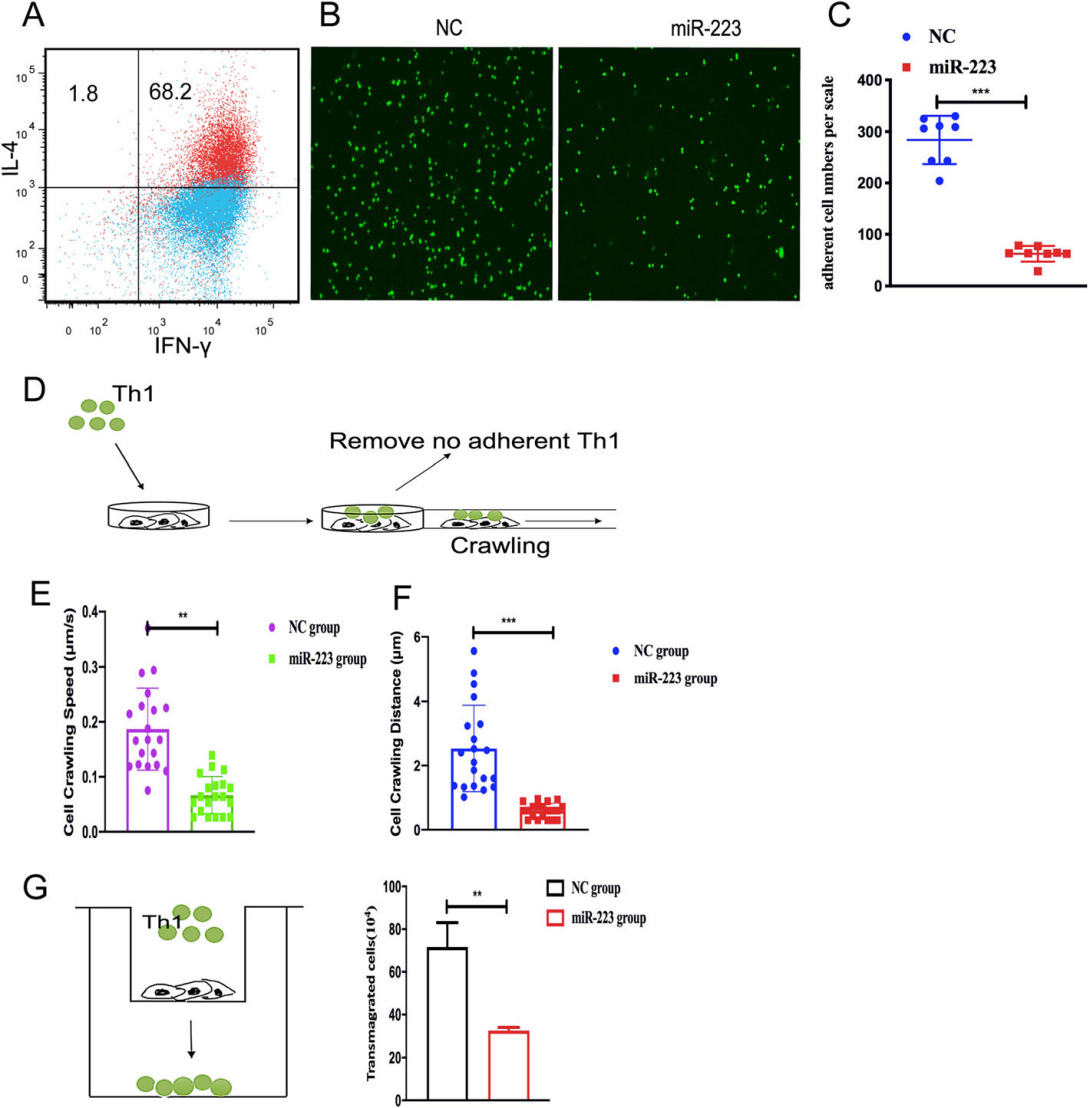
MiR-223 impaired T cell crawling, adhesion, and transmigration in vitro*.* Measurement of CD4^+^ Th1 differentiation using flow cytometry (**a**). Adhesion assay for the Th1 cells stained with green CellTracker™ and co-cultured with mLECs transfected miR-223 mimics (100 nM) or NC for 45 min (**b**). The average number of green adherent cells in transfected miR-223 group showed significant decrease (**c**). Crawling assay for the Th1 cells stained with green CellTracker™ seeded on the mLEC transfected miR-223 mimics or NC for 20 min. Time-lapse imaging was performed on Operetta CLS™ (**d**). Compare with NC group, there was significant decrease in the crawling speed and distance of T cells in miR-223 mimics group (**e**, **f**). The transmigration assay results indicated the number of Th1 cells stained with green CellTracker™ in miR-223 group was significantly lower compared with that of normal control (**g**). Data are presented as mean ± SEM. Measured in two independent experiments. **P < 0.01, ***P < 0.001

Several studies indicated that ICAM-1 in the vascular endothelium was associated with T cell migration, and ICAM-1 expression was upregulated in lymphatic vessels and blood vessels in an inflammatory microenvironment, which promoted T cell migration and inflammatory responses [31–33]. Based on the inhibition of *ICAM-1* expression mediated by miR-223, we further evaluated whether miR-223 could affect T cell migration. After Th1 cell induction, the proportion of CD4 cells differentiated into Th1 cells was up to 68.2% (Fig. 4a). The green CellTracker™-labeled Th1 cells were co-cultured with mLECs transfected miR-223 mimics (100 nM) or normal control for 45 min. The results of fluorescence microscope showed that the average numbers of green CellTracker™ adherent cells in transfected miR-223 group significantly decreased (Fig. 4b, c, *P* < 0.001). Concomitantly, the crawling experiment results showed that miR-223 significantly inhibited the migration speed and distance of Th1 cells compared with the negative control group (Fig. 4d–f and Supplementary video 1 and 2, *P* < 0.01, *P* < 0.001). Moreover, we assessed whether miR-223 could regulate ICAM-1 to change the extravasation of Th1 cells. Th1 cells stained with green CellTracker™ were co-cultured with mLECs transfected miR-223 mimics or NC in transwell system for 4 h. The number of Th1 cells in the miR-223 group was significantly lower than that of the NC group (Fig. 4g, *P* < 0.01). These results suggested that miR-223 could restrain the migration and adhesion of Th1 cells through ICAM-1.

### MiR-223 infusion remarkably inhibited the pathogenesis of aGvHD in mice

**Fig. 5:**
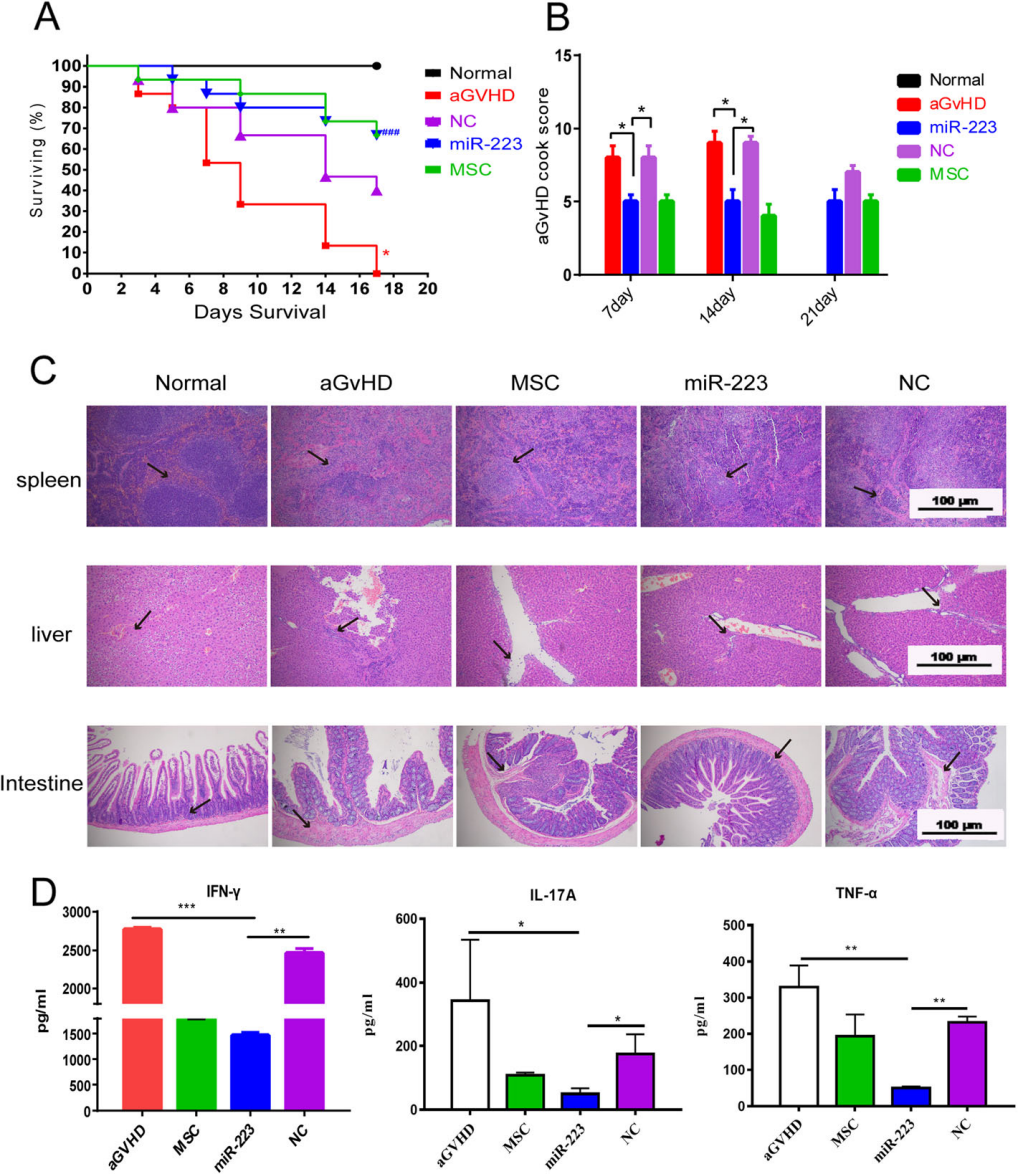
MiR-223 infusion remarkably inhibited the development of aGvHD. Recipient BABL/6 mice were irradiated (8.0 Gy) and intravenously injected with C57BL/6j bone marrow cells plus splenocytes. The survival rate of miR-223 group was further improved compared with the other groups (**a**). The clinical aGvHD scores of miR-223 group of mice were significant alleviated compared with the scores of the aGvHD and NC groups of mice (**b**). For the representative pathologic changes on day 14, miR-223 infusion dramatically decreased lymphocyte infiltration in the GvHD target tissues, including the spleen, liver, and intestine (**c**). The inflammatory cytokine level (IFN-γ, IL-17a, and TNF-α) showed significant decline in the blood serum of the miR-223 group compared with that of the aGvHD or NC (**d**). Data were presented as mean ± SEM. Measured in three independent experiments, *n* = 5 per group. *P < 0.05, **P < 0.01, ***P < 0.001

We further investigated whether miR-223 could inhibit T cell migration to relieve aGvHD symptoms. Our data showed that miR-223Agomir improved the general condition of aGvHD mice and reduced mortality between days 5 and 17 (Fig. 5a). On day 7 and day 14, mice from miR-223Agomir group presented significant lower aGvHD clinical scores than those from NC group and aGvHD group (Fig. 5b). These data indicated that miR-223 could reduce aGvHD symptoms of mice.

Then we investigated the therapeutic effects of miR-223 on organs and tissues in aGvHD mice using hematoxylin-eosin staining to determine the pathological changes and infiltration of inflammatory cells in spleen, liver, and intestine. After miR-223Agomir treatment, spleen, liver, and intestine damage were attenuated. The tissue structure remained relatively intact, while the inflammatory cell infiltration declined (Fig. 5c). The inflammatory improvement in aGvHD mice caused by miR-223 was further assessed by measuring the expression of proinflammatory factors in serum. The expression of IFN-γ, IL-17A, and TNF-α was lower in the miR-223Agomir-treated group (1468.4 ± 59.8, 49.78 ± 16.8, and 49.9 ± 4.5) than that in the aGvHD group (2773.3 ± 28.4, 342.8 ± 192.2, and 329.2 ± 60.3) or negative control group (2463.1 ± 59.9, 174.3 ± 62.3, and 232.3 ± 15.6) (Fig. 5d). These findings indicated that miR-223 attenuated the pathological damages in tissues and reduced the expression of proinflammatory factors in aGvHD mice.

### MiR-223 reduced migration of donor T cell to recipient spleens

**Fig. 6:**
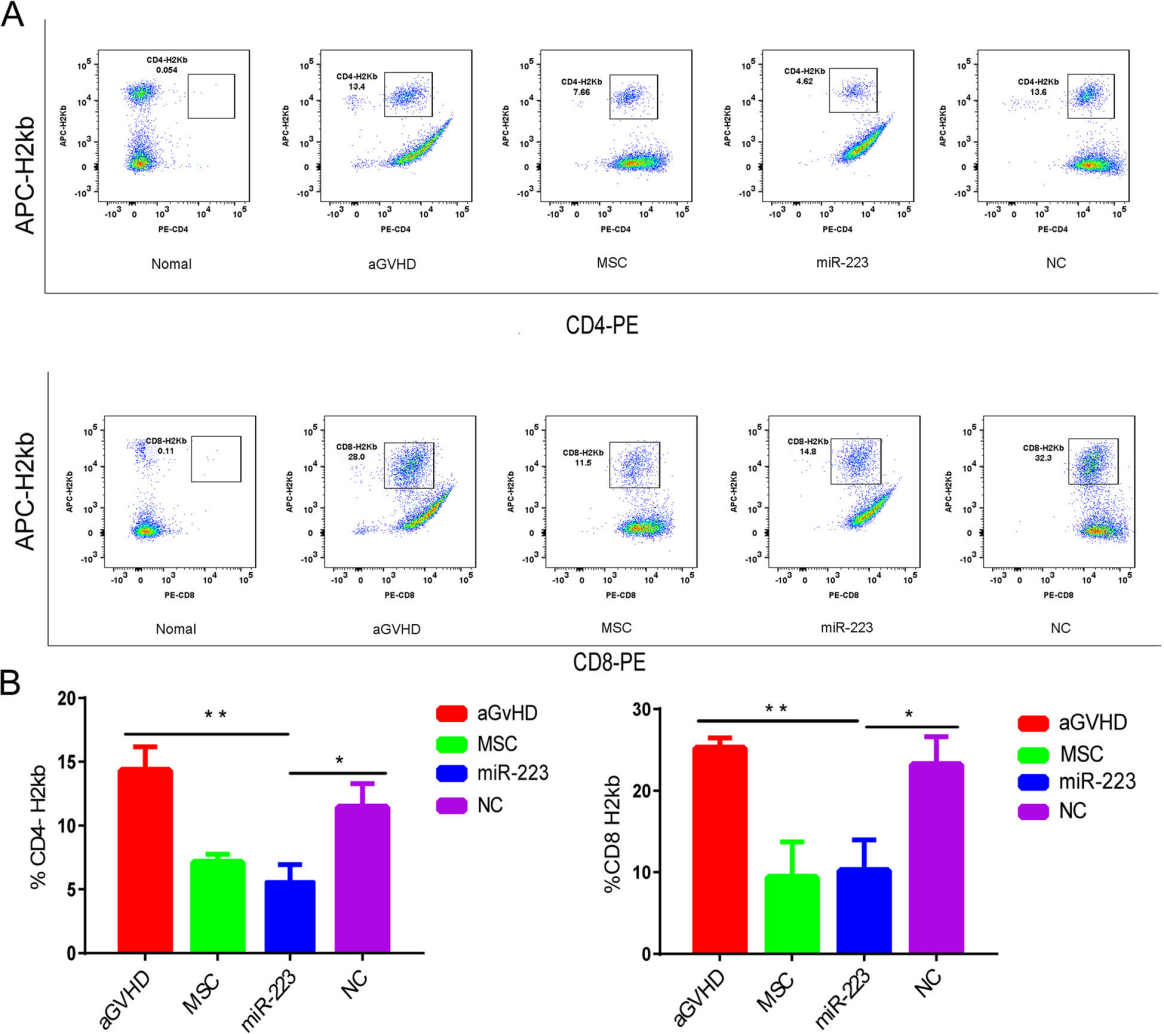
MiR-223 reduced donor T cell migration to recipient mice spleens. The proportion of H2kb ^+^ CD4 and CD8 T cell in recipient mice spleens on day 7 was assessed by flow cytometry. Compared with NC and aGvHD group, H2kb ^+^ CD4 and H2kb ^+^ CD8 cell percentages in miR-223 group showed significant reduction (**a**). The average number of H2kb ^+^ CD4 and H2kb ^+^ CD8 cells in miR-223 group showed significant decline (**b**). Data were presented as mean ± SEM. Measured in three independent experiments, *n* = 7 per group. *P < 0.05, **P < 0.01

The pathogenesis and progression of aGvHD were considered to be associated with activation and subsequent expansion of donor T cells in recipient secondary lymphoid organs (SLOs) [34]. These cells then migrated to peripheral target organs to cause tissue damage by cell-mediated cytotoxicity. Flow cytometry results showed that the percentage of splenic H2kb^+^ CD4 T cell and H2kb^+^ CD8 T cell was lower in the miR-223 infusion recipient mice compared with that of the aGvHD mice and negative control group (Fig. 6a). The average number of H2kb ^+^ CD4 and H2kb ^+^ CD8 cells showed significant decrease in miR-223 group, which was similar to that of the MSC group (Fig. 6b). These data further implicated the role of miR-223 in MSCs mediated immunosuppression in vivo. The effects of miR-223 on MSCs were likely to be associated with reducing migration of donor T cells to recipient mice.

### MiR-223 reduced donor T cell homing to recipient tissues and organs

**Fig. 7:**
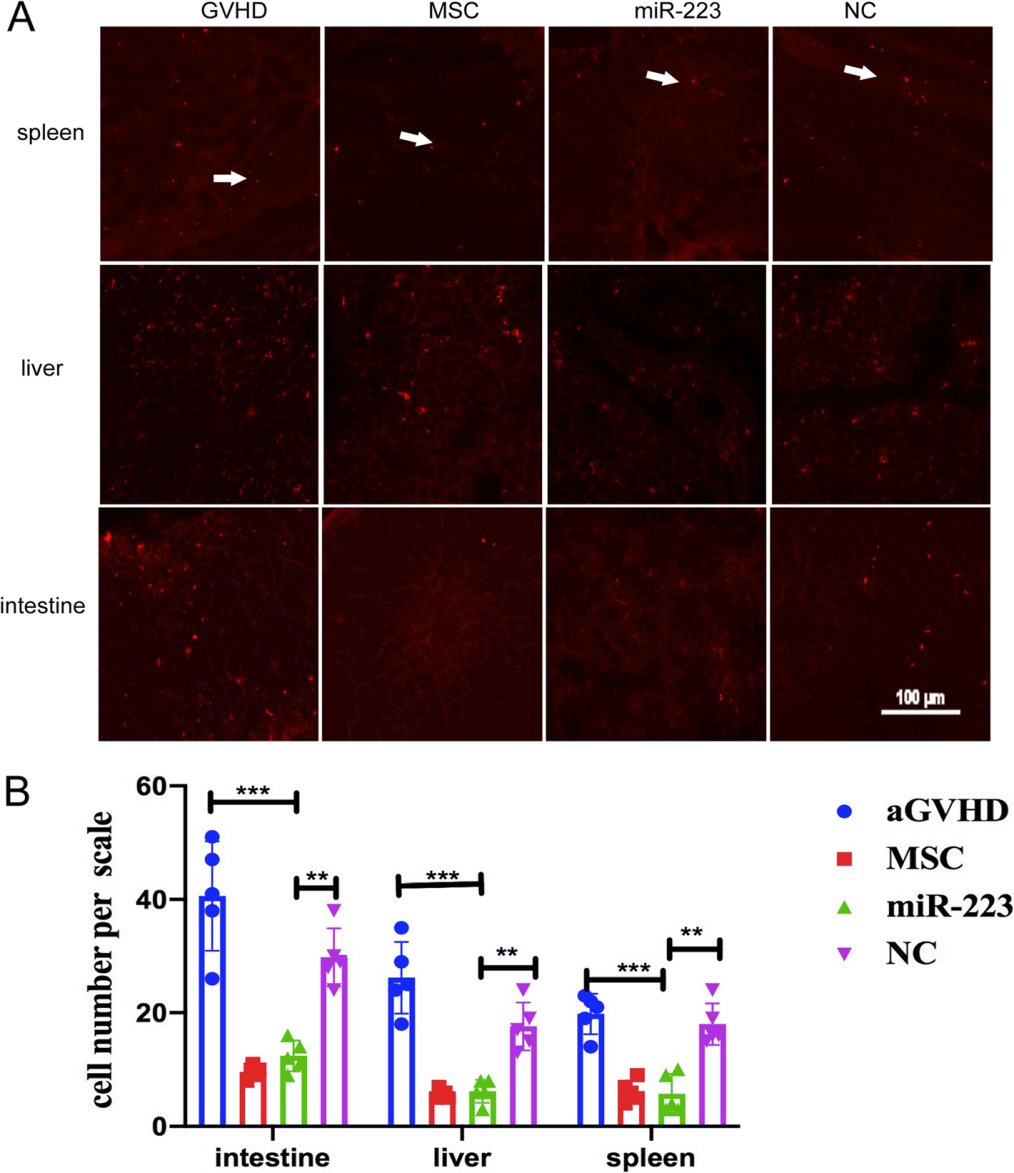
MiR-223 reduced donor T cells homing to recipient tissues and organs. The red CM-dil-labeled splenic T cells and bone marrow cells were transplanted into aGvHD mice. On day 3, the CM-dil-labeled splenic T cells migrated to recipient mice spleen, liver, and intestine in vivo*.* Compared with NC and aGvHD group, significant decline was noticed in the CM-dil-labeled splenic T cells in miR-223 group (**a**). Compared with NC and aGvHD group, the average number of red CM-dil-labeled cells in spleen, liver, or intestine showed a significant decline in miR-223 group (**b**). Five fields were randomly in 5 mice. Data were presented as mean ± SEM. Measured in three independent experiments, *n* = 5 per group. *P < 0.05, **P < 0.01, ***P < 0.001

To further determine the roles of miR-223 in modulating the allogeneic T cell homing, we transplanted CM-dil-labeled splenocytes and bone marrow cells into aGvHD mice. Three days after transplantation, we observed red CM-dil-labeled splenocytes in the spleens, livers, and intestines of each group by a fluorescence microscope. Compared with that of the miR-223 group, significant increase was noticed in the number of red splenocytes in the negative control (*P* < 0.01, Fig. 7) and aGvHD group (*P* < 0.001, Fig. 7). Moreover, we also evaluated the ICAM-1 expression after miR-223Agomir infusion to aGvHD with immunohistochemical staining. The results indicated ICAM-1 expression in miR-223 group are decreased comparing with aGvHD and MSC group (Fig.S2). These suggested that miR-223 inhibited allogeneic T cell infiltration into the tissues and organs of recipient mice.

## Discussion

MSCs are considered as a promising candidate for stem cell therapy for its immunosuppressive properties [5]. Previous studies demonstrated that MSCs had different immunosuppressive functions in different inflammatory microenvironment, depending on the type and concentration of inflammatory factors causing different effects for aGvHD [4–6]. Our study showed that miR-223 derived from MSCs-Exo attenuated the progression in the mice aGvHD model. MiR-223 treatment had effects on pathological lesions. Meanwhile, it could reduce the donor T cell migration and homing in vivo. In vitro experiments showed that treatment with MSCs-Exo derived miR-223 directly caused reduction in the adhesion and migration of T cells by inhibiting ICAM-1 expression. These studies suggested that MSCs-Exo-miRNAs present similar immunoregulation function to MSCs, which can directly affect the progression of aGvHD.

Some investigations highlighted that the migration of the effector T cells to the target tissues was crucial for the pathogenesis of aGvHD [35, 36]. Nowadays, MSCs have been employed as efficient treatment options for aGvHD. However, MSCs used for aGvHD treatment were mainly administered through intravenous transplantation, which may result in a possibility of remaining in the blood-rich tissues (e.g., liver, lung, and spleen) [1, 6]. Therefore, methods for enhancing homing to the target organ and anti-inflammatory effects of MSCs are urgently needed to improve their clinical efficacy. Accumulating evidence indicated that MSCs could secret exosomes containing cytokines, chemokines, and microRNAs [15, 16]. MSCs-Exo showed immunosuppressive effects on peripheral blood monocytes, T cells, B cells, and NK cells [15, 37]. The effects were depending on the number of exosomes absorbed by T cells [37]. Moreover, several studies demonstrated that MSCs-Exo derived miRNAs exerted immunosuppressive capacities [14, 38, 39]. These experiments demonstrated that MSCs-Exo-derived miRNAs could regulate inflammatory processes and display therapeutic potential. However, the elucidation of molecular mechanisms of MSCs-Exo-derived miRNA action in aGvHD remains unclear. In this study, we first reported the MSCs-Exo-derived miRNA expression spectrum from mb-MSCs and huc-MSCs using high-throughput sequencing. According to the miRNA profile of MSCs-Exo, we found that miR-223 was highly expressed in mb-MSCs-Exo and huc-MSCs-Exo. Furthermore, MSCs were transplanted into C57BL/6j mice, and the serum miR-223 expression showed increase. Here, our data revealed that miR-223 is from MSC-derived exosome.

Previous evidence demonstrated that MSCs attenuated aGvHD through altered migratory properties of T cells and DCs [40, 41]. However, the specific mechanisms are not well defined. We found that *ICAM-1* was one of the target genes of miR-223, which was identified through a dual-luciferase reporter gene assay. ICAM-1 could interact with lymphocyte function-associated antigen 1 (LFA-1) to mediate lymphocyte migration, membrane penetration, and activation [42–44]. We further showed that miR-223 inhibited *ICAM-1* expression on HUVECs and lymphatic endothelial cells, which decreased the T cell migration, the penetration of vascular barriers, and adhesion to lymphatic vessels. ICAM-1 was expressed on the myeloid lineages, most likely DCs, and could interact with LFA-1, which expressed on T cells, to support the high-velocity migration of T cells. In our study, MSCs-Exo containing miR-223 could inhibit ICAM-1 expression, which restrains this interaction effect, thus reduced donor T cell migration. However, whether the expression of LFA-1 changes needs further investigated.

To determine whether the effects of miR-223 on T cells were beneficial to aGvHD treatment, we established the mouse aGvHD model. Then the model was treated with miR-223Agomir after bone marrow transplantation, which showed that miR-223 improved the general status in aGvHD mice and simultaneously alleviated lymphocyte infiltration and destruction of the liver, lungs, and other tissues. In vivo donor lymphocyte homing results were similar to in vitro experiments. Specifically, the donor T cell migration to the spleen, liver, intestine, and lungs in the miR-223 group was lower than that in the other groups. In addition, the effects of miR-223 on T cells also alleviated aGvHD inflammation as the expression of serum IFN-γ, TNF-α, and IL-17 showed significant reduction.

Unlike the previous studies, our study focused on the homing of donor T cells to target tissues. Our data showed that miRNAs derived from MSCs-Exo may contribute to the immunosuppressive activity of transplanted MSCs in the in vivo environment of aGvHD. Normally, aGvHD occurred upon migration of donor lymphocytes to tissues and organs in the recipient, such as liver, intestine, and spleen, which then disrupted the normal function of these organs. Lymphocytes may migrate to secondary lymphoid organs for further maturation and activation, which then led to secretion of proinflammatory cytokines causing subsequent an inflammatory storm and tissue damages. This inflammatory infiltration and destruction thereby induce aGvHD, leading to transplant failure and even death [1, 45].

MiR-223 is one of MSCs-Exo containing numerous miRNAs, which attenuate aGvHD through restrained donor T cell migration, other miRNAs maybe a great threat for the aGvHD. MSCs also released other immunosuppressive molecules, growth factors, exosomes, chemokines, complement components, and various metabolites. In our study, we demonstrated that miR-223Agomir could attenuate the aGvHD progression by reducing donor T cell migration and decreased inflammatory cytokines, but the clinical scores and the survival results are lower to compare with MSCs group, which indicated MSCs could exert a greater threat in aGvHD. Further investigation into the underlying molecular mechanisms is needed to allow more effective guidance of MSCs function for clinical applications.

In summary, we found that miR-223 derived from MSCs-Exo showed similar biological function with the MSCs. MiR-223 infusion remarkably inhibited the progression of aGvHD in mice. MiR-223 restrained T cell adhesion and migration via inhibiting the expression of ICAM-1 on both lymphatic and vascular endothelial cells in vitro. Meanwhile, it reduced infiltration by donor lymphocytes on organs and tissues and alleviated inflammation in aGvHD mice. On this basis, we concluded that miR-223 derived from MSCs-Exo alleviated murine aGvHD development, which would be promising in treating aGvHD. Moreover, little is known about the mechanisms underlying MSCs-Exo-derived miRNAs mediated suppression of aGvHD. Our studies demonstrated that MSCs-Exo-derived miR-223 reduced the progression of aGvHD through restraining donor T cell migration. Additional studies are necessary to obtain profound understanding on this phenomenon and promote its clinical utilization.

## Conclusion

Our work showed a new role for MSCs-Exo-derived miRNA in immunoregulation and the therapeutic potential of aGvHD. In support of this, we constructed MSCs-Exo miRNAs profile of huc-MSCs-Exo and mb-MSCs-Exo using high-throughput sequencing. We also found MiR-223 derived from MSCs-Exo could inhibit ICAM-1 expression and restrain adhesion and migration of T cells in vitro. Moreover, miR-223Agomir could attenuate the aGvHD symptoms by reducing T cell migration and inflammatory response. On this basis, we concluded that miR-223 derived from MSCs-Exo could alleviate murine aGvHD development.

## Supplementary Information

**Additional file 1: Figure S1.** Identification of murine compact bone mesenchymal stem cells derived exosomes. Nucleated cells isolated from compact bones of mice displayed fibroblast-like morphology (A). The adherent cells were stained with Wright-Giemsa (B). Multilineage differentiation potential was assessed by inducing adipogenic or osteogenic capacities in vitro. Adipogenic differentiation was indicated by the presence of lipid drops stained with oil red O (C, D). Osteogenic differentiation was shown by intracytoplasmic accumulation of alkaline phosphatase (E, F). Immunophenotyping of culture-expanded adherent cells from murine compact bones-derived adherent cells was analyzed using flow cytometry (G). The mb-MSCs-Exo (marked by the black arrows) was observed under electron microscopy (H). NanoSight analysis indicated that particle size of MSCs-Exo was 30–100 nm (I). Exosome-specific markers (e.g. TSG101 and CD63) were identified by Western blot (J).

**Additional file 2: Figure S2.** ICAM-1 expression in recipient mice spleen detected by Immunohistochemical staining.

**Additional file 3: Supplementary Video 1.** The video of green CellTracker™ labeled Th1 cells in the crawling assay for the miR-233.

**Additional file 4: Supplementary Video 2.** The video of green CellTracker™ labeled Th1 cells in the crawling assay for the normal control.

**Additional file 5.**

**Additional file 6.**

## Data Availability

The datasets used and/or analyzed during the current study are available from the corresponding author on reasonable request.

## Abbreviations

MSCs: Mesenchymal stem cells
aGvHD: Acute graft-versus-host disease
huc-MSCs: Human umbilical cord MSCs
mb-MSCs: Murine compact bone MSCs
MSC exosomes: MSCs-Exo
qPCR: Quantitative real-time PCR
mLECs: Mouse lymphatic endothelial cells
ALP: Alkaline phosphatase
HUVECs: Human umbilical vein endothelial cells
