# Supplementary figures and images for: Mesenchymal stem cell exosome-derived miR-223 alleviates acute graft-versus-host disease via reducing the migration of donor T cells
Source: Stem Cell Res Ther. 2021 Feb 26;12:153. doi: 10.1186/s13287-021-02159-2 (PMC7913292; doi:10.1186/s13287-021-02159-2)

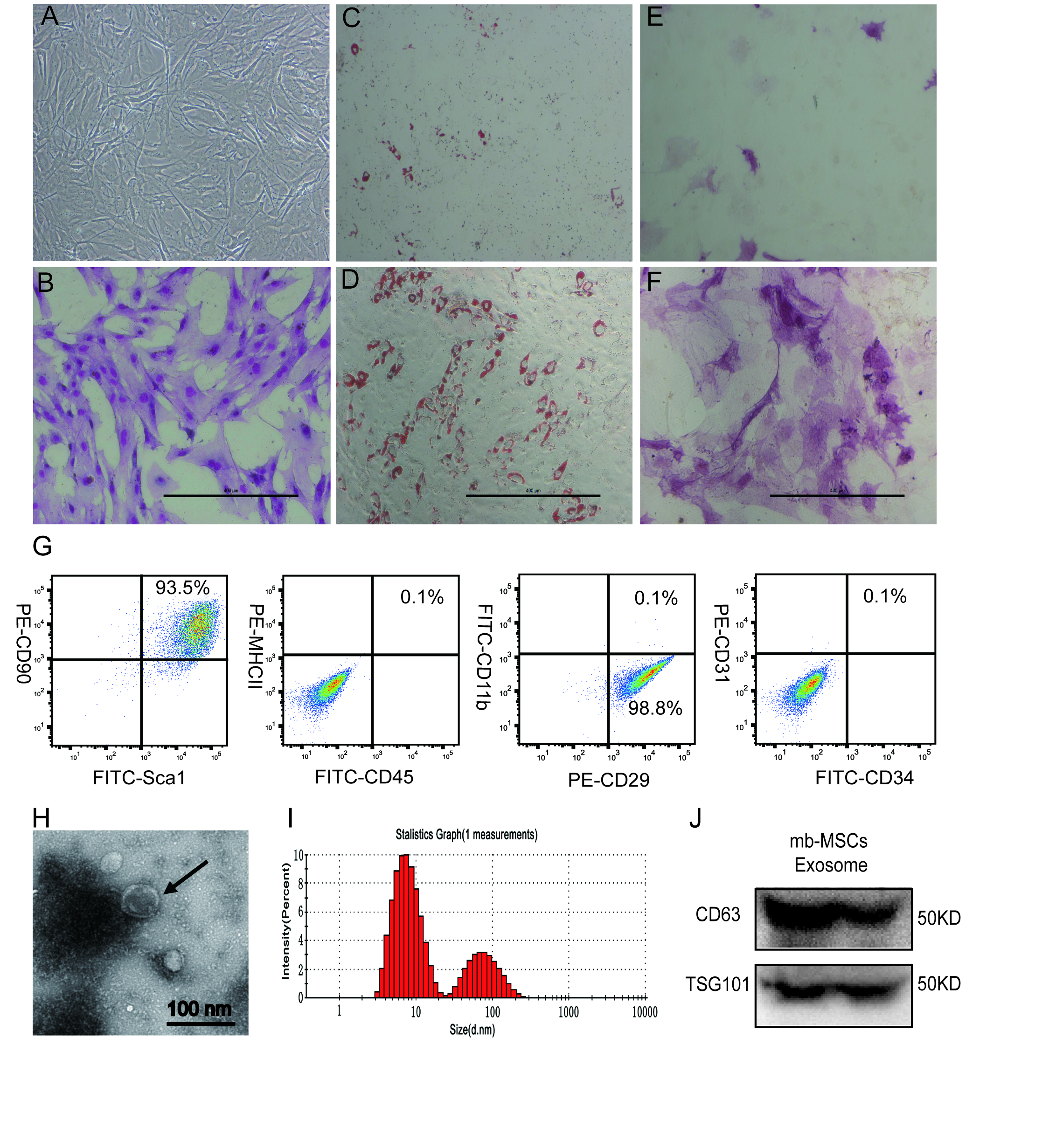

Supplement: Supplementary file 1 — Additional file 1: Figure S1. Identification of murine compact bone mesenchymal stem cells derived exosomes. Nucleated cells isolated from compact bones of mice displayed fibroblast-like morphology (A). The adherent cells were stained with Wright-Giemsa (B). Multilineage differentiation potential was assessed by inducing adipogenic or osteogenic capacities in vitro. Adipogenic differentiation was indicated by the presence of lipid drops stained with oil red O (C, D). Osteogenic differentiation was shown by intracytoplasmic accumulation of alkaline phosphatase (E, F). Immunophenotyping of culture-expanded adherent cells from murine compact bones-derived adherent cells was analyzed using flow cytometry (G). The mb-MSCs-Exo (marked by the black arrows) was observed under electron microscopy (H). NanoSight analysis indicated that particle size of MSCs-Exo was 30–100 nm (I). Exosome-specific markers (e.g. TSG101 and CD63) were identified by Western blot (J). [file 13287_2021_2159_MOESM1_ESM.tif]

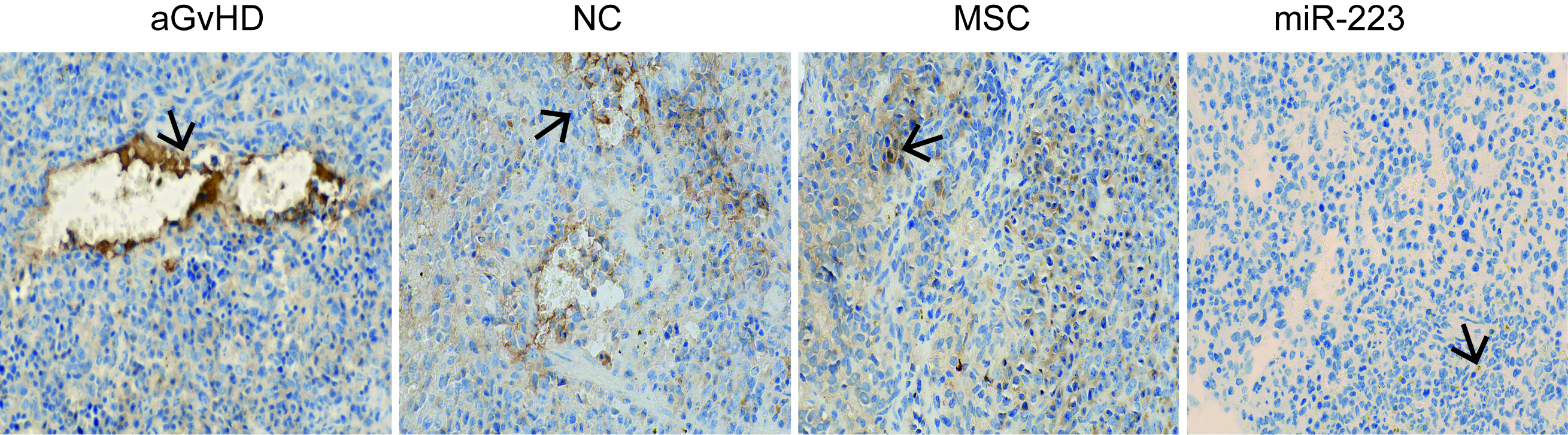

Supplement: Supplementary file 2 — Additional file 2: Figure S2. ICAM-1 expression in recipient mice spleen detected by Immunohistochemical staining. [file 13287_2021_2159_MOESM2_ESM.tif]
